# The Processing of Symbolic and Nonsymbolic Ratios in School-Age Children 

**DOI:** 10.1371/journal.pone.0082002

**Published:** 2013-11-29

**Authors:** Gaëlle Meert, Jacques Grégoire, Xavier Seron, Marie-Pascale Noël

**Affiliations:** Psychological Sciences Research Institute, Université catholique de Louvain, Louvain-la-Neuve, Belgium; The University of Western Ontario, Canada

## Abstract

This study tested the processing of ratios of natural numbers in school-age children. Nine- and eleven-year-olds were presented collections made up of orange and grey dots (i.e., nonsymbolic format) and fractions (i.e., symbolic format). They were asked to estimate ratios between the number of orange dots and the total number of dots and fractions by producing an equivalent ratio of surface areas (filling up a virtual glass). First, we tested whether symbolic notation of ratios affects their processing by directly comparing performance on fractions with that on dot sets. Second, we investigated whether children’s estimates of nonsymbolic ratios of natural numbers relied at least in part on ratios of surface areas by contrasting a condition in which the ratio of surface areas occupied by dots covaried with the ratio of natural numbers and a condition in which this ratio of surface areas was kept constant across ratios of natural numbers. The results showed that symbolic notation did not really have a negative impact on performance among 9-year-olds, while it led to more accurate estimates in 11-year-olds. Furthermore, in dot conditions, children’s estimates increased consistently with ratios between the number of orange dots and the total number of dots even when the ratio of surface areas was kept constant but were less accurate in that condition than when the ratio of surface areas covaried with the ratio of natural numbers. In summary, these results indicate that mental magnitude representation is more accurate when it is activated from symbolic ratios in children as young as 11 years old and that school-age children rely at least in part on ratios of surface areas to process nonsymbolic ratios of natural numbers when given the opportunity to do so.

## Introduction

Ratios are frequent in our daily lives: they are used for cooking, plumbing, sales and probabilities, to name but a few areas. They establish a relation of division between either two discrete magnitudes (i.e., ratios of natural numbers; e.g., the ratio between the number of women and the total number of members within an executive committee) or two continuous magnitudes (e.g., ratios of surface areas). There are many natural situations in which ratios are experienced by animals or human beings, such as when animals look for food [1,2] or when babies perceive the aspect ratio of objects or people around them [3]. Ratios are also an important part of the school curriculum, which includes learning symbolic notation of ratios in the form of fractions (e.g., “1/4”). So far, research has not yet fully investigated the processing of ratios of natural numbers in school-age children despite the contrast between the acknowledged complexity of learning fractions at school and the amazing ability of infants to process nonsymbolic ratios of natural numbers. The present study addresses this gap (1) by testing the impact of symbolic notation on processing of ratios in school-age children and (2) by testing whether children’s performance on nonsymbolic ratios of natural numbers relies at least in part on ratios of surface areas that often covary with the former ratios. 

### Research on Nonsymbolic Ratios of Natural Numbers

Research on the processing of numbers and more particularly of natural numbers (i.e., positive whole numbers) has led researchers to suggest that numerical magnitudes are mentally represented in an approximate way along an analogue continuum that obeys the Weber-Fechner law [4 - 7]. According to this law, performance in numerical tasks depends on the ratio of magnitudes (called the *ratio effect*) instead of their absolute difference. For instance, in a magnitude-comparison task, performance is poorer when the ratio of magnitudes is closer to 1 (e.g., when comparing 7 and 9) than when this ratio is further from 1 (e.g., when comparing 2 and 3). 

Recent findings have suggested that the magnitude of ratios of natural numbers could be mentally represented in an approximate way and that these representations are supported by the same mental approximate number system as that used for the representation of the magnitude of natural numbers [8]. For instance, infants as young as six months are able to discriminate two ratios in terms of their magnitude and this ability is apparently limited in the same way as for natural numbers [9]. Thus six-month-olds are able to discriminate between sequences of different numbers of sounds when the ratio of these absolute magnitudes is 2 (e.g., 16 vs. 8 sounds) but not when that ratio is 1.5 (12 vs. 8 sounds) [10]. In a similar way, they are able to discriminate between two ratios when the ratio of these ratios is 2 (e.g., they can discriminate that the numerical relation between 1 PacMan and 2 pellets differs from that between 1 PacMan and 4 pellets) but not when that ratio is 1.5 (e.g., they cannot discriminate 1 PacMan/2 pellets from 1 PacMan/3 pellets) [9]. Furthermore, the *ratio effect*, which is well-known for the comparison between two natural numbers, has been shown to apply when children aged from five to seven compare a set transformed by a scaling factor (1/2 or 5/2) to a second set [11,12]. 

Nevertheless, at school-age, children seem to struggle with ratios of natural numbers. Jeong, Levine and Huttenlocher [13] asked 6-, 8- and 10-year-olds to compare ratios in the context of a game. Children had to select among two game boards the one that would give them the greater probability of winning. Game boards were made up of two parts that were either continuous (continuous condition) or discretized into units (discrete condition). The probability of winning was determined by the ratio between the favourable part (gain) and the unfavourable part (no gain). All children succeeded in the task for the continuous condition. In contrast, for the discrete condition, 6-year-olds performed at the level of success by chance and 8- and 10-year-olds succeeded only when ratios were relatively distant. When ratios were closer, these children tended to rely on the number of units in the favourable part without considering its relation to the number of units in the unfavourable part or in the whole. 

The children’s trend to omit the numerical relation linking the ratio components has been frequently reported at primary school age [e.g., 13, 14, 15]. This bias has been labeled the *whole number bias* [16] as it apparently results from the tendency to apply the knowledge of positive whole numbers (i.e., natural numbers) to ratios without taking their specific properties into account. This bias appears when children are asked to compare or match ratios between two sets but has not been demonstrated when children are asked to match this kind of ratio to a ratio of surface areas [17]. As this bias is specific to the case where both ratios are between sets, Boyer, Levine and Huttenlocher [17] have suggested that it does not reflect any trouble with processing ratios of natural numbers per se but it would reflect activation of inappropriate counting strategies when the nature of stimuli gives children the opportunity to use them. 

One limit of these studies in school-age children is that processing of nonsymbolic ratios of natural numbers has not been tested while controlling for the covariance of ratios of surface areas with ratios of natural numbers. In these studies, children’s performance may have relied, partially or fully, on the ratio of the cumulative surface areas occupied by the items. Indeed, if children are presented a bar discretized into three equal parts, two of which are orange and the last one is grey, and they process the ratio between the orange part and the whole, they can rely either on the ratio between the orange surface area and the total surface area, on the ratio between the number of orange parts and the total number of parts, or on both ratios since they are both equal to 2/3. This issue is important to address as it is acknowledged that preschoolers’ performance relies at least in part on continuous magnitudes when they are asked to judge the number of items in a set [18] and school-age children may do the same [19]. The same conclusion could be true for ratios, all the more that processing ratios between continuous magnitudes might be less challenging for school-age children than processing ratios of natural numbers [13]. In addition, the processing of ratios of surface areas might have been fostered in the previous studies [13,17] by the fact that the ratio components were continuous parts discretized into units (e.g., a bar or a donut discretized into units) instead of sets of independent items (e.g., sets of dots). 

The present study tested the processing of ratios between two sets that were made up of independent items and contrasted a condition where the ratio of surface areas covaried with the ratio of natural numbers with a condition where it was kept constant across ratios of natural numbers. It allowed us to test whether children’s estimates increased with ratios of natural numbers even when the ratio of surface areas was kept constant and, more importantly, whether performance was poorer in that condition than when the ratio of surface areas covaried with the ratio of natural numbers.

### Research on Symbolic Ratios

Behavioural and functional MRI studies have shown that, when adults are asked to process the magnitude of symbolic ratios (i.e., fractions), they access a mental approximate representation of their magnitude [20 - 29]. Children are also able to do this, at least at a certain stage of learning. Meert et al. [30] asked 10- and 12-year-old children to compare fractions with common denominators (e.g., 3/7 and 5/7) and fractions with common numerators (e.g., 2/3 and 2/5). Their performance depended on the numerical distance between fractions indicating that they accessed an approximate representation of the magnitude of fractions (see also 31). Iuculano and Butterworth [32] showed that 10-year-old children were also able to estimate positions that corresponded to fractions on a number line.

Like the processing of nonsymbolic ratios, the processing of fractions is affected by the whole number bias [33-38]. For instance, when children begin to learn fractions, they tend to think erroneously that the larger the components, the larger the numerical value of the fraction [38]. Research on the processing of fractions in adults has suggested that they also use strategies relying on the components in magnitude-comparison tasks but in a more appropriate way than children do [21,24,26,39]. For instance, they rightly compare denominators when all the fraction pairs have common numerators [e.g., 39] and they tend to compute cross products for fractions that have different numerators and denominators and that are close on the number line [21]. 

The impact of symbolic notation on the processing of ratios of natural numbers has been recently tested in adults. Meert, Grégoire, Seron and Noël [40] asked adults to estimate the magnitude of symbolic ratios (fractions) and of nonsymbolic ratios (ratios between the number of orange dots and the total number of dots). The results showed that symbolic notation allowed adult participants to generate a more precise mental representation of ratio magnitudes than nonsymbolic notation even when denominators were as large as 29 (e.g., 7/29). This advantage of the symbolic notation had been already suggested for natural numbers [41]. 

The present study tested the impact of symbolic notation in school-age children at two different stages of familiarity with this notation (in 9- and 11-year-olds). For the first time, children’s performance on fractions was directly compared with their performance on nonsymbolic ratios. This contrast allowed us to test whether symbolic notation had a positive or negative impact on the processing of ratios of natural numbers in these age groups. 

### The Present Study

The present study investigated the processing of ratios of natural numbers in school-age children. For that purpose, a magnitude-estimation task, which was adapted from that used by Meert et al. [40] with adults, was presented to children at two different stages of familiarity with fractions. Nine- and 11-year-olds (Grades 4 and 6 respectively) were shown fractions and sets made up of orange and grey dots. They were asked to estimate the magnitude of fractions and of the ratio between the number of orange dots and the total number of dots by producing an equivalent ratio of surface areas. Production of ratios of surface areas from estimation of ratios of natural numbers had the advantage of not favouring the whole number bias and hence allowed the processing of ratios of natural numbers to be investigated independently of this bias. It was expected that, in such a task, school-age children’s estimates would increase consistently with ratio magnitudes.

In order to test whether children’s performance relies on ratios of surface areas when they are asked to process ratios of natural numbers, two conditions were contrasted. They had to estimate part-whole ratios for sets of either heterogeneous or homogeneous dots. In the heterogeneous-dot condition, size of dots varied so that the ratio between surface area occupied by orange dots (the critical part) and surface area occupied by all the dots (the whole) was constant across all ratios of natural numbers. Accordingly, participants could not perform the task correctly by processing ratios of surface areas but had to process ratios of natural numbers. Conversely, in the homogeneous-dot condition, all the dots were the same size, so that ratios of surface areas covaried with ratios of natural numbers. We hypothesized that, if school-age children’s performance relies at least in part on ratios of surface areas when they are asked to process ratios of natural numbers, their estimates should be less variable and more accurate when ratios of surface areas covary with ratios of natural numbers (homogeneous-dot condition) than when it do not (heterogeneous-dot condition). 

In order to test impact of symbolic notation, performance on fractions was compared with performance on heterogeneous-dot sets (nonsymbolic condition in which children were obliged to process ratios of natural numbers to succeed in the task as the ratio of surface areas was controlled for). We hypothesized that impact of symbolic notation depends on the degree of familiarity with it and hence on participants’ age and on numerical size of components (fractions with small denominators are learned first and are more frequent). In the initial stages of the learning of fractions, a process that is acknowledged to be slow and complex [e.g., 16, 38], symbolic notation may impede the processing of ratio magnitudes. In later stages, as experience of fractions increases, we hypothesized that symbolic notation activates a more precise mental representation of magnitude than the nonsymbolic format does (as seen in adults [40]). Accordingly, we expected estimates to be less accurate and more variable for fractions than for heterogeneous-dot sets, especially among 9-year-olds and for fractions with large components (which are learned later and are less frequent). We also expected an improvement of performance due to symbolic notation to appear for fractions, at least for those with small denominators, in 11-year-olds. 

## Methods

### Ethical Statement

Ethical approval was gained from the Ethics Committee of the Psychological Sciences Research Institute at the Université catholique de Louvain (Belgium) under the reference Projets2011/GM-1. Written informed consent was obtained from parents prior to testing. Verbal consent was also obtained from children themselves. 

### Participants

A total of 17 children in Grade 4 (mean age = 9:4 years old, Min – Max = 8:9 – 10:2; 5 boys) and of 19 children in Grade 6 (mean age = 11:3 years old, Min – Max = 10:9 – 12:1; 9 boys) took part in the study in return for a gift voucher for a book. They were all attending regular classes at two schools in the French-speaking Community of Belgium and were tested during the autumn term. 

According to the curriculum used in the Belgian French-speaking Community’s education system, children aged 8 to 10 (Grades 3 to 5) learn (a) to divide a figure or set into parts and verbally express the fraction corresponding to one part, (b) to read and write fractions with denominators up to 10, (c) that taking a fraction of a figure or set involves two successive operations (dividing it into the number of parts indicated by the denominator and taking the number of parts indicated by the numerator), (d) to place fractions on a graduated number line, (e) to express equivalence between two ratios with a different total number of parts (up to 10) and (f) to express fractions that correspond to whole figures or sets. Children aged 10 to 12 (Grades 5 to 6) learn (a) to read and write fractions with denominators up to 100 and (b) to express equivalence between two ratios with a different total number of parts (up to 20).

### Stimuli and Design

Participants estimated the magnitude of fifteen ratios. These ratios resulted from the combination of five ratio magnitudes and three denominator sizes (see Table 1). Ratio magnitudes were ~ .22, ~ .40, ~ .53, ~ .65 and ~ .75. Exact ratio magnitudes deviated from these approximate magnitudes by a maximum of .03. The denominator was small (less than or equal to 5), medium (between 6 and 9) or large (between 20 and 29). Each magnitude was thus represented by three ratios that varied in the size of the denominator (e.g., ~ .40 was represented by 2/5, 3/8 and 11/28). All the ratios were irreducible in order to prevent participants from simplifying.

**Table 1:** Overview of the ratios to be estimated.

	**Ratio magnitude**				
**Denominator Size**	**~ .22**	**~ .40**	**~ .53**	**~ .65**	**~ .75**
Small (≤ 5)	1/4 (.25)	2/5 (.40)	1/2 (.50)	2/3 (.67)	3/4 (.75)
Medium (from 6 to 9)	2/9 (.22)	3/8 (.38)	5/9 (.56)	5/8 (.63)	7/9 (.78)
Large (from 20 to 29)	7/29 (.24)	11/28 (.39)	13/25 (.52)	17/26 (.65)	16/21 (.76)

Note. Five levels of approximate ratio magnitude were used. The exact magnitude of each ratio is in brackets.

**Figure 1:**
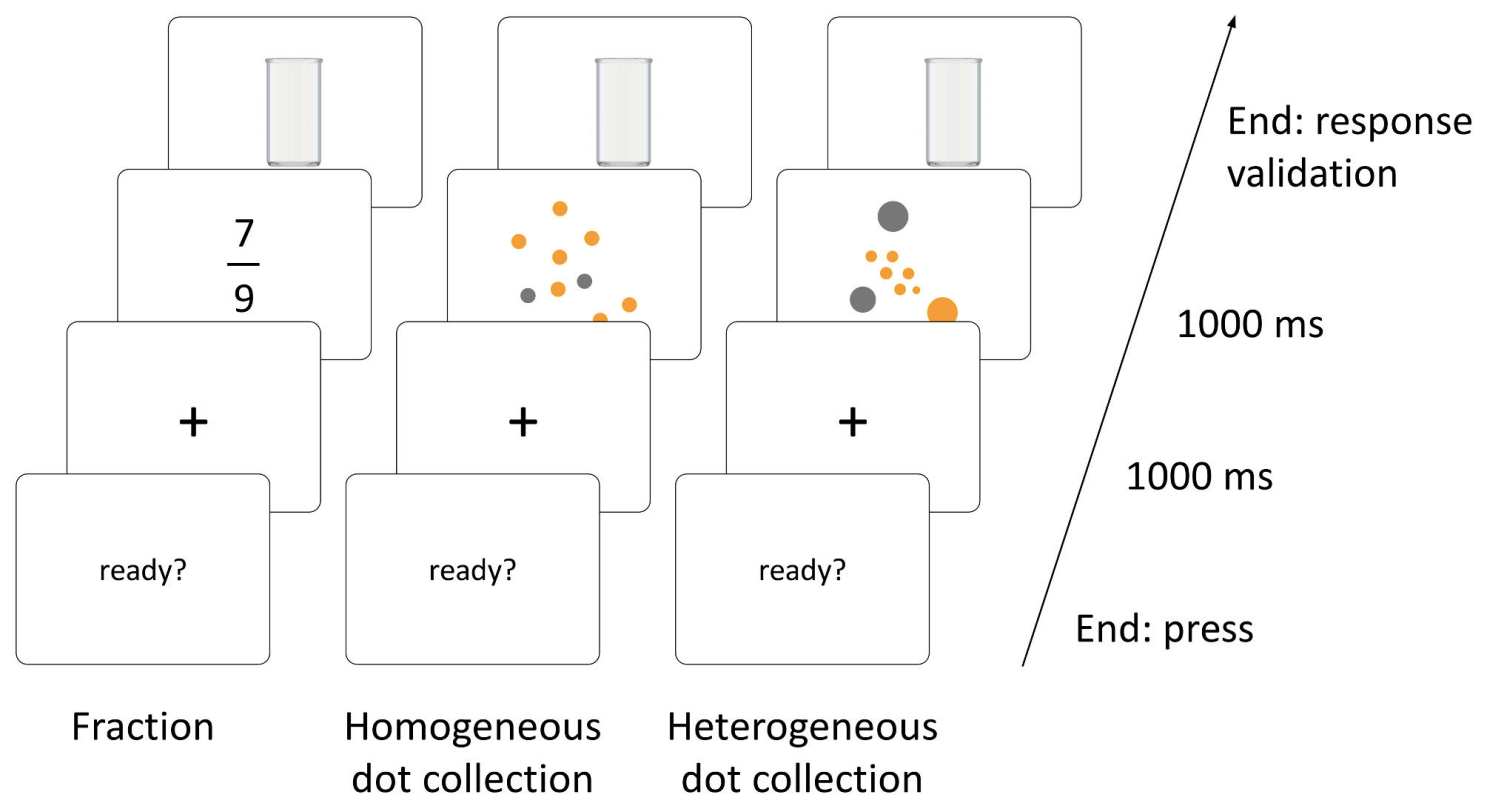
Instance of stimulus for each ratio format and time course of a trial. Participants were asked to estimate fractions as well as the ratio between the number of orange dots and the total number of dots. Sets were made up of either homogeneous dots (without controlling for the ratio of surface areas) or heterogeneous dots (with such a control). Participants responded by filling a glass so that the ratio (water surface/total surface) was equal to the estimated ratio. The time line represents the time course of a trial.

Ratios were presented to each participant in three formats: (1) symbolic format (fractions), (2) nonsymbolic format without controlling for ratios of surface areas (homogeneous-dot sets) and (3) nonsymbolic format with such a control (heterogeneous-dot sets) (see Figure 1). 

Fractions were made up of Arabic numerals presented in black printed characters (Arial font, normal) on a white screen. The numerator was presented above the denominator with the fraction bar between them. The height and the width of fractions were respectively 3.3° and 1.9° assuming a viewing distance of 60 cm. 

For both nonsymbolic formats, sets of orange and grey dots were used. The ratio to be estimated was that between the number of orange dots (the critical part) and the total number of dots (the whole) (e.g., for 2/5, 2 orange dots out of 5 dots). Dots were centred and grouped inside an invisible circle with a diameter of 13.8°. The interdot distance was at least equivalent to one dot radius. In the homogeneous-dot condition, all dots had a radius of 1°, so that the ratio of surface areas (the orange surface area/the surface area occupied by all dots) covaried with the ratio of natural numbers. In the heterogeneous-dot condition, eight sizes of dot were used (between 0.5° and 2° with an increment of 0.2°), so that the orange surface area was the same as the grey surface area and the size difference between the smallest and the largest dot was maximized within both subsets (i.e., orange and grey dots). Accordingly, the ratio between the orange surface area and the total surface area was 1/2 for each ratio of natural numbers (controlling for the ratio of surface areas) and the size of dots did not covary with their number (as would have been the case if we had used constant dot size within each subset). Eight different configurations were created by Matlab for each numerical ratio.

### Procedure

Children were tested individually. They were asked to estimate the magnitude of part-whole ratios represented by fractions and dot sets by producing equivalent ratios of surface areas in a virtual glass. To do this, they used a potentiometer that allowed them to virtually adjust the water level in the glass so that the ratio of surface areas between water and the whole glass was equivalent to fractions or ratios of dot sets. Participants were told that their responses were not expected to be completely accurate as the task involved estimation but they were asked to do their best and to respond as fast as possible. Furthermore, participants were asked to estimate fractions without performing exact calculations and to estimate the ratio between the number of orange candies and the total number of candies without counting (dots were presented as candies in order to make the task more entertaining). Instructions specified that a candy was always equal to 1, whatever its size.

To begin a trial, participants had to press the key of the response box. A fixation cross appeared on the computer screen for 1000 ms, followed by a fraction or a dot set for 1000 ms (see Figure 1). Next, an empty glass appeared and participants had to use the potentiometer in the response box to adjust the water level in the glass. The potentiometer allowed them to flash images of the glass with different levels of water onto the screen so that the water level appeared to increase when the potentiometer was turned in the clockwise direction and to decrease when it was turned anticlockwise. Participants were asked to press the key of the response box once the water level matched their estimate. E-Prime 1.1 recorded the number of levels filled in the glass (from 1 to 254) and this was divided by 254 to get the ratio. The potentiometer had a relatively fast speed (1/2 turn = 1 glass) and a relatively slow speed (1 turn = 1 glass) but did not have either a starting or a stopping point. The use of two speeds and the absence of starting and stopping points prevented participants from using the degree of turning or visual marks on the potentiometer. They could only estimate the water level in the glass visually. 

A block was created for each format in which the 15 ratios were presented eight times (four with the fast potentiometer speed and four with the slow speed). Accordingly, the estimation task was made up of 360 trials (3 formats x 15 ratios x 8 repetitions). Within a block, all ratios were randomly presented once, half the ratios with the fast potentiometer speed and the other half with the slow speed before the next presentation of the 15 ratios began. 

The block order varied between participants according to a Latin square design. There were two sessions separated by several days. In the first session (60 min), children were invited to help a cartoon character to collect three stars in order to get a cookery certificate. They were told that, to win a star, a task had to be performed on ratios that are commonly used when cooking. Once children accepted the invitation, the concept of estimation was explained as “processing magnitudes quickly and approximately, that is without counting, computing or measuring”. To illustrate this instruction, a picture of an apple basket was then flashed on the computer screen and children were asked to estimate the number of apples. It was then explained to them that ratios can also be estimated for stimuli such as a set of shapes (squares and circles) or a portion of pie. Next, participants familiarized themselves with the potentiometer by filling the virtual glass and then emptying it. Finally, they performed two experimental blocks. Each block was introduced by showing, in a booklet, the estimate that might be produced in the glass for 1/3 and 21/23, by showing the time course of two trials of the computerized task and by inviting children to perform eight training trials. No feedback was given during either training or experimental blocks. In the second session (lasting 30 min), children performed the remaining block of the magnitude-estimation task after they had again familiarized themselves with the potentiometer. 

### Dependent Variables and Statistical Analyses

For each ratio presented in each format, we computed the mean and the standard deviation (SD) of the eight estimates as well as the mean absolute error score (AES). SD is a measure of the consistency of estimates around the *subjective* magnitude (i.e., the mean of the eight estimates given by the participant for a given ratio). AES is a measure of the deviation of estimates from the *objective* magnitude that does not take into account the direction of the deviation (i.e., whether ratios were under- or overestimated). This variable was computed by subtracting the estimate from the ratio magnitude for each trial in each participant and by taking the absolute value of this difference. This absolute difference was averaged across the eight repetitions of the same ratio in order to get the mean AES. A score of 0.10 means that, on average, estimates deviated from the ratio magnitude by 0.10. For instance, if AES equals to 0.10 for 3/4 (0.75), it means that estimates were on average around 0.65 or 0.85. Finally, we computed the mean error score (ES) that indicates the direction of the deviation in order to check that the classical pattern of biases (i.e., overestimation below the half and underestimation above the half) was found in the present study. Results for this variable are presented in supplementary materials (Results S1 and Figure S1) as they were not directly related to the main issues addressed by the present study. 

The .05 significance level was used for all analyses. P values were corrected using the Huynh-Feldt formula if the sphericity assumption was violated. 

## Results

### Children’s Ability to Deal with the Task: Analyses on Mean Estimates

In order to test whether estimates increased consistently with ratio magnitudes, linear mixed models were run with mean estimates as the dependent variable and ratio magnitudes as the fixed effect for each age group and each format. These models included a random intercept and a random slope for participants as these factors significantly contributed to the variance according to the likelihood ratio test (see Table 2). The slope was positive and significant irrespective of the format and the age group (see Table 3). This result indicates that estimates globally increased with ratio magnitudes for dot sets (even when ratios of surface areas were controlled for) and for fractions and hence that 9 and 11-year-olds could deal with the task requirement. 

**Table 2:** Results of likelihood ratio tests comparing the fitting of the model including both the intercept and the slope as random factors with the fitting of the model including only the intercept.

**Age**	**Format**	**-2 RLL (Intercept**)	**-2 RLL (Intercept + Slope**)	**Df**	**χ^2^**
9	Fractions	-356.87	-366.34	2	9.47*
	Homog. dots	-463.56	-473.97	2	10.41*
	Heterog. dots	-431.69	-474.41	2	42.72*
11	Fractions	-617.78	-627.40	2	9.62*
	Homog. dots	-695.45	-729.64	2	34.19*
	Heterog. dots	-702.63	-746.89	2	44.26*

Note. * *p* < .01, -2 RLL = -2 Restricted Log Likelihood

The unstructured covariance matrix was used for the model including both the intercept and the slope as random factors in order to allow these factors to covariate. The variance component matrix was used for the model including the intercept as a random factor.

**Table 3:** Results of the linear mixed models run on the participants’ responses with the ratio magnitude as a predictor according to the age group and the ratio format.

				**Intercept**				**Slope**			
**Age**	**Format**	**AIC**	**BIC**	***EST***	***SE***	***df***	***t***	***EST***	***SE***	***df***	***t***
9	Fractions	-358.34	-344.21	0.12	0.03	16	3.70*	0.75	.06	16	12.92*
	Homog. dots	-465.97	-451.84	0.11	0.03	16	3.61*	0.82	.05	16	17.50*
	Heterog. dots	-466.41	-452.27	0.17	0.04	16	4.19*	0.73	.07	16	10.42*
11	Fractions	-627.40	-604.82	0.05	0.02	18	2.68*	0.87	.04	18	24.45*
	Homog. dots	-721.64	-707.06	0.04	0.02	18	1.96	0.94	.04	18	22.42*
	Heterog. dots	-746.89	-724.30	0.09	0.02	18	3.86*	0.88	.04	18	19.74*

Note. * *p* ≤ .01, AIC = Akaike’s Information Criterion, BIC = Schwarz’s Bayesian Criterion, EST = Estimates of fixed effects, SE = Standard Error, *df* = degree of freedom, *t* = value of *t* test

Spence [42] and Hollands and Dyre [43] showed that power models also fit well to mean estimates of ratio magnitudes because these models take the pattern of bias (under- and overestimation) into account. We did not fit these models to our data because the pattern of bias was beyond the aim of the present study and the design was thus not created for testing these models. Power models require the whole range of the magnitudes between 0 and 1 to be covered and a great number of different magnitudes to be presented, since specific predictions are made for each portion of the range of ratio magnitudes. The present study used only five magnitudes with the smallest and the largest one being far from the boundaries 0 and 1. Nevertheless, we ran an ANOVA on the error score in order to explore whether the classical pattern of biases shown in adults was also found in school-age children (see Results S1 and Figure S1).

### Impact of Ratios of Surface Areas and of Symbolic Notation: ANOVAs on AES and on SD

Next, we tested (1) whether children’s estimates of nonsymbolic ratios of natural numbers relied at least in part on ratios of surface areas when given the opportunity to do so and (2) whether symbolic notation of ratios led to more accurate estimates, at least in 11-year-olds and for fractions with small denominators. An ANOVA was run on AES with Format (three levels: fractions, heterogeneous dots and homogeneous dots) and Denominator Size (three levels: small, medium and large) as repeated factors and Age Group (two levels: 9- vs. 11-year-olds) as a between factor. Post hoc tests were run to explain any significant main effect or interaction. The critical comparison for assessing the impact of ratios of surface areas was the comparison of performance on homogeneous-dot sets (no control for ratios of surface areas) with that on heterogeneous-dot sets (such a control), whereas the critical comparison for assessing the effect of symbolic notation was the comparison of performance on fractions with that on heterogeneous-dot sets (condition in which children had to process ratios of natural numbers due to the control for ratios of surface areas). 

The effect of Age Group was significant, *F*(1, 34) = 16.05, *p* < .01. Estimates deviated more from the objective magnitude in 9-year-olds (*M* = .12, *SD* = .03) than in 11-year-olds (*M* = .08, *SD* = .02). The effect of Format was not significant, *F*(2, 68) = 2.29, *p* > .10, but the effect of Denominator Size, *F*(1.26, 42.82) = 6.82, *p* < .01, and the interaction between Format and Denominator Size, *F*(3.14, 106.66) = 7.79, *p* < .01, were significant. All the interactions that involved Age Group were not significant [Format * Age Group: *F*(2, 68) = 1.65, *p* > .10; Denominator Size * Age Group: *F*(1.26, 42.82) = 2.70, *p* = .10; Format * Denominator Size * Age Group: *F*(3.14, 106.66) < 1, *p* > .10]. 

**Figure 2:**
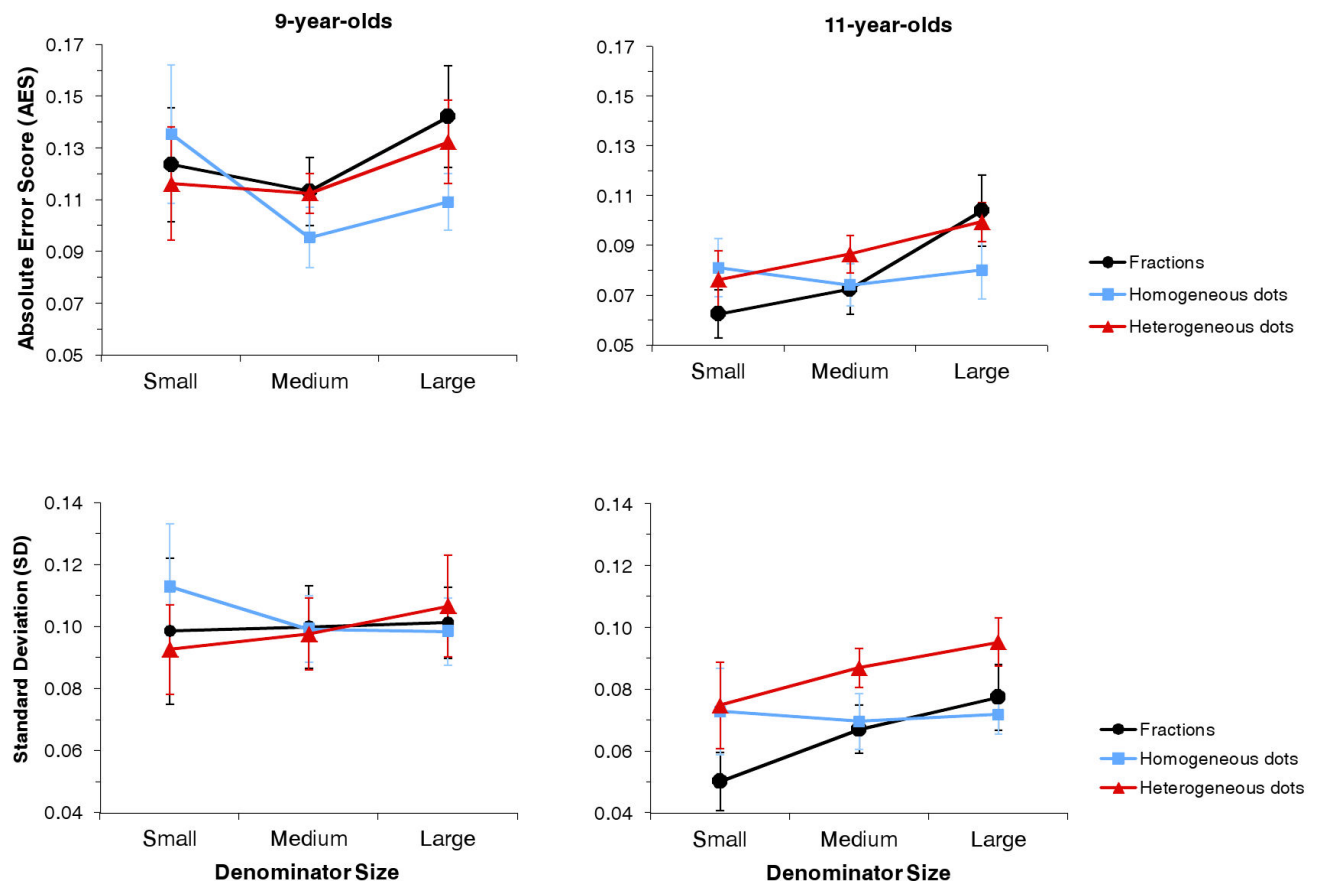
The mean absolute error score (top panel) and the mean standard deviation (bottom panel) according to the denominator size and the ratio format by age group. Error bars represent the 95% confidence intervals corrected according to the method suggested by Cousineau [44] for repeated measures.

Despite the absence of significant triple interaction, the pattern of results for 9-year-olds differed from the one for 11-year-olds as shown in Figure 2. The absence of interaction might be explained by the fact that the task appeared challenging for 9-year-olds and that AES was quite variable in this group. Because running analyses on the two groups combined masked some effects, we looked at results separately for each age group. The interaction between Format and Denominator Size was significant in 9-year-olds, *F*(4, 64) = 2.80, *p* = .05, and in 11-year-olds, *F*(4, 72) = 8.97, *p* < .01.

In 9-year-olds, the effect of Format was not significant for ratios with small denominators, *F*(2, 32) < 1, *p* > .10, but it was significant for ratios with medium, *F*(2, 32) = 4.17, *p* = .04, and large denominators, *F*(2, 32) = 5.19, *p* = .01. The homogeneous-dot condition led to lower AES (i.e., more precise estimates) than the heterogeneous-dot condition for ratios with medium, *t*(16) = –3.51, *p* < .01, and large denominators, *t*(16) = –2.92, *p* = .01. Furthermore, AES for fractions did not differ significantly from AES for heterogeneous-dot sets for ratios with medium, *t*(16) = –0.12, *p* > .10, and large denominators, *t*(16) = –0.76, *p* > .10, but it was higher than AES for homogeneous-dot sets for these ratios [medium denominators: *t*(16) = –2.07, *p* = .06; large denominators: *t*(16) = –3.26, *p* < .01]. In summary, 9-year-olds’ estimates relied at least in part on ratios of surface areas in the homogeneous-dot condition when sets were beyond the subitizing range and symbolic notation led to estimates as accurate as estimates of nonsymbolic ratios of natural numbers (i.e., heterogeneous dot condition). 

In 11-year-olds, the effect of Format was significant for all the sizes of denominators [small: *F*(2, 36) = 6.92, *p* < .01; medium: *F*(2, 36) = 3.74, *p* = .03; large: *F*(2, 36) = 4.61, *p* = .03]. Regarding the impact of ratios of surface areas, the same result as in 9-year-olds appeared: AES for homogeneous-dot sets was similar to AES for heterogeneous-dot sets for ratios with small denominators but it was lower than for heterogeneous-dot sets for ratios with medium, *t*(18) = –2.93, *p* < .01, and large denominators, *t*(18) = –3.31, *p* < .01. Concerning the impact of symbolic notation, when denominators were small, AES was lower for fractions than for heterogeneous-dot sets, *t*(18) = 2.82, *p* = .01, and homogeneous-dot sets, *t*(18) = 4.28, *p* < .01. When denominators were medium, AES for fractions was lower than AES for heterogeneous-dot sets, *t*(18) = 2.16, *p* = .05, but did not differ from AES for homogeneous-dot sets, *t*(18) < 1, *p* > .10. When denominators were large, processing fractions was as accurate as processing heterogeneous-dot sets, *t*(18) < 1, *p* > .10, but less accurate than processing homogeneous-dot sets, *t*(18) = –2.33, *p* = .03. These results indicate a gain in accuracy due to symbolic notation when denominators were small or medium at the age of 11.

In summary, 11-year-olds produced more accurate estimates than 9-year-olds, whatever the format. In both age groups, estimates were more accurate in the homogeneous-dot condition than in the heterogeneous-dot condition when sets were beyond the subitizing range. This result indicates that children relied at least in part on ratios of surface areas to perform the task, when given the opportunity to do so. Regarding the impact of symbolic notation, an emergent gain in accuracy due to this notation was shown at the age of 11: AES for fractions was smaller than AES for heterogeneous-dot sets for ratios with small and medium components in this age group. 

The same analyses as the ones performed on AES were run on SD (see Results S2 for detailed results). SD and AES correlated in a highly significant and positive way for the three formats (fractions: *r* = .89, *p* < .01; homogeneous dots: *r* = .81, *p* < .01; heterogeneous dots: *r* = .73, *p* < .01) and results were quite similar for these variables (see Figure 2). In 9-year-olds, no evidence of an impact of the format on the variability of estimates was found. In 11-year-olds, controlling for ratios of surface areas (i.e., using heterogeneous-dot sets) for medium and large sets but not for small ones led to more variability than when this control was not applied (i.e., homogeneous-dot sets). Furthermore, in this age group, symbolic notation led to less variable estimates compared with the heterogeneous-dot condition regardless of the denominator size. Finally, estimates were less variable in 11-year-olds than in 9-year-olds for fractions and for homogeneous-dot sets. 

## Discussion

The present study investigated the processing of ratios of natural numbers in school-age children. More particularly, we tested the impact of ratios of surface areas and of symbolic notation on that processing in two age groups. Nine- and 11-year-olds were asked to estimate part-whole ratios presented in the form of dot sets (either homogeneous or heterogeneous dots) and fractions by producing an equivalent ratio of surfaces areas. This design allowed us to investigate the processing of ratios independently of the whole number bias. This bias, which refers to children’s trend to consider one part of the ratio independently of the other part or the whole, has been shown in tasks involving only ratios of natural numbers [13,17]. This bias has not been shown for a task in which ratios of natural numbers had to be processed in relation to ratios of surface areas [17].

### Processing of Nonsymbolic Ratios of Natural Numbers

Regarding the processing of part-whole ratios applied to dot sets, we first hypothesized that school-age children’s estimates would increase consistently with ratio magnitudes even if ratios of surface areas are controlled for (i.e., in the heterogeneous-dot condition). This hypothesis was drawn from the fact that, when ratios between continuous magnitudes are controlled for, even sixth-month-olds are able to discriminate ratios of natural numbers if these ratios are sufficiently far apart [9]. Our results showed that, at the group level, the 9- and 11-year-olds’ estimates globally increased with the magnitude of the ratio between the number of orange dots and the total number of dots even when they could not rely on ratios of surface areas. This result extends the result reported by Boyer, Levine and Huttenlocher [17] to stimuli for which ratios of surface areas were controlled for. These authors showed that school-age children were able to match ratios of natural numbers to ratios of surface areas. However, ratios of natural numbers were not controlled for ratios of surface areas, which allowed participants the opportunity to rely on these ratios as was the case in the homogeneous-dot condition of the present study. 

It is noteworthy that, in the present study, children could not have succeeded in the task by only processing the magnitude of the critical part or by separately processing the magnitude of the part and the whole. As the size and the nature of the whole varied between dot sets and the virtual glass, participants could not have matched directly the magnitude of the critical subset (the set of orange dots) to the water surface area. Participants had to process the part-whole ratio in order to proportionally adjust the level of water to the magnitude of the whole glass. 

Furthermore, we have to qualify the claim that children used estimation to perform the task because, for ratios with small denominators (smaller or equal to five), subitizing might have allowed children to identify the magnitude of the part and the whole in an accurate way and might even lead children to symbolize them by verbal labelling. This might have contributed to more accurate estimates of ratios. It remains nevertheless possible that estimation was used to process ratios because exact computation seems unlikely at this age (this would involve dividing the numerator by the denominator in order to get a decimal number) and not helpful for the present task (producing an approximate ratio of surface areas). We could also wonder whether counting might have been used to identify the part and the whole for ratios with medium and large denominators. It seems unlikely because counting was explicitly discouraged by instructions and because the duration of presentation of dot sets (one second) was too short to separately count the critical part and the whole, especially for children. Moreover, counting is completely irrelevant for processing the magnitude of the ratio itself.

Our results showed that, despite the global trend of estimates to increase with actual magnitudes, estimates significantly deviated from them and in a greater extend in 9-year-olds than in 11-year-olds. This indicates that the processing of nonsymbolic ratios of natural numbers is still in progress at the age of 9. The improvement between the age of 9 and 11 may be due to the refinement of mental numerical representations as children develop [e.g., 45, 46] and/or more efficient access to mental magnitude representations from ratios. The systematic instruction given at school in ratios, multiplication and division may play a role in these improvements. As a design very similar to the one of the present study was used in a previous study in adults [40], we could compare 11-year-olds’ estimates to adults’ estimates. The absolute error score did not significantly differ between these groups, suggesting that ability to estimate nonsymbolic ratios of natural numbers is already mature at the age of 11. 

We next investigated whether school-age children’s estimates relied at least in part on ratios of surface areas when they were asked to estimate ratios of natural numbers. When the denominator was beyond the subitizing range, the participants performed better in the homogeneous-dot condition (i.e., when ratios of surface areas covaried with ratios of natural numbers) than in the heterogeneous-dot condition (i.e., when these ratios did not covary). This result indicates that their performance relied at least in part on ratios of surface areas. This is reminiscent of data suggesting that young children’s performance relies at least in part on continuous magnitudes that are correlated with the number of items in a collection when they are asked to judge this number [18,19, see also 47]. The same propensity to rely on ratios of surface areas has been reported in adults as well [40].

It is noteworthy that children’s trend to rely on ratios of surface areas might have been fostered by response modality. Children were asked to produce ratios of surface areas. Therefore, the degree of stimulus-response compatibility was greater in the homogeneous-dot condition than in the heterogeneous-dot condition. The production of ratios of surface areas with a fixed whole area and a high range of possible responses (254 levels in the virtual glass) was chosen as a response modality because of its advantage and the limits of the alternative response modalities (such as producing fractions or blackening a subset of the whole set). The production of ratios of surface areas had the advantage of making the use of counting and exact computation fully irrelevant for the task and it allowed us to design a task that did not favour the whole number bias. Furthermore, this response format best matches assumptions about the nature of mental magnitude representations due to its continuous property and seems thus the best alternative to measure variability and accuracy of these representations.

Among the two dot conditions, the homogeneous-dot condition was the most similar one to those used in previous studies [e.g., 13, 15, 17, except 9]. By contrasting the homogeneous-dot condition and the heterogeneous-dot condition, we showed that children tended to rely on ratios of surface areas when given the opportunity to do so even if ratio components were sets made up of independent entities (instead of continuous parts discretized into units such as in [13,17]). Therefore, future studies aiming to investigate the processing of ratios of natural numbers per se should control for this confounded variable. In addition, results of previous studies should be considered in the light of the present result: children might have relied on ratios of surface areas to perform the task all the more that stimuli were continuous magnitudes that had been discretized into units [13,15,17]. However, the absence of control for ratios of surface areas does not call into question the main conclusion of these studies: school-age children tend to ignore the link between the ratio components (the whole number bias) in tasks involving only ratios of natural numbers, probably because discrete magnitudes activate counting strategies that are inappropriate to the task.

### Processing of Symbolic Ratios

Turning to the processing of symbolic ratios, the first important result is that, at the group level, estimates of fractions globally increased with the magnitude of fractions in both groups of school-age children. This result was shown with a set of fractions including some fractions that had not yet been covered in the school curriculum (i.e., fractions with denominators larger than 10 for 9-year-olds and larger than 20 for 11-year-olds). It suggests that even 9-year-olds have some sense of how to process the magnitude of fractions even though understanding fractions is a slow and gradual process [e.g., 16, 38]. So far, studies that have shown school-age children’s ability to mentally represent the magnitude of fractions have been carried out with children from 10 to 13 years old [31,32] or with children selected for their good performance in a magnitude-comparison task on fractions [30]. The present study has found this ability among younger children without any selection of participants and using fractions that were not all included in the curriculum even if we should notice that estimates deviated from the actual magnitude in a significant way and in a greater extent in 9-year-olds than in 11-year-olds. 

The children’s ability to represent fraction magnitudes does not necessarily imply that these representations were directly activated from fractions. Indeed, they might as well be activated by first processing the magnitude of the numerator and the denominator and then their numerical relation. It seems not plausible that school-age children are able to directly represent the magnitude of all fractions since there are an infinite number of fractions to represent the same magnitude and most of them are not familiar, especially for children. We might hypothesize that a direct mapping can take place for some fractions, the most familiar ones, after frequent exposures. Future studies should test this issue, firstly in adults. 

To test the impact of symbolic notation on processing of ratios, we compared performance on fractions with that on heterogeneous-dot sets. We expected poorer performance for fractions than for heterogeneous-dot sets, especially in 9-year-olds and for fractions with medium and large denominators. We hypothesized that as children’s experience with fractions increases, symbolic notation would allow school-age children to activate a more precise mental magnitude representation than the nonsymbolic format allows them to do. The results showed that the 9-year-olds’ estimates were globally as accurate and as variable for fractions as they were in the heterogeneous-dot condition. By 11 years of age, in comparison with the heterogeneous-dot condition, symbolic notation was associated with a substantial gain in accuracy for ratios with denominators up to 9 and with a significant reduction in variability for fractions with denominators up to 29. Using a very similar paradigm, Meert, Grégoire, Seron and Noël [40] showed that, in adults, improvement in performance due to symbolic notation was even greater as it appeared for fractions with denominator up to 29 in comparison with both the heterogeneous- and the homogeneous-dot conditions. 

These results suggest that symbolic notation does not really have a negative impact on the processing of ratios in 9-year-old children but it already has a positive impact on performance by the age of 11. These results are consistent with the hypothesis that the approximate mental representation of numbers is more precise when it is activated from symbols than from sets [41]. We might hypothesize two sources of better precision for the mental representation of fraction magnitudes. The mental representation of the magnitude of the components may be more precise when it is activated from symbols (compared to when it is activated from sets) and hence the processing of their numerical relation would lead to a more precise representation of the fraction magnitude. We might also hypothesize that direct mapping takes place between some fractions (the most familiar ones; e.g., ½ and ¼) and the mental representation of their magnitude and that this mapping allows these representations to be activated more efficiently from symbolic ratios than from nonsymbolic ones. Future studies should test whether such a mapping exists in adults and when it appears across development. 

## Conclusions

The present study has deepened our knowledge of the processing of ratios in school-age children. First, we have shown that 9- and 11-year-olds’ estimates of nonsymbolic ratios of natural numbers tend to rely on ratios of surface areas when given the opportunity to do so. Second, the present study has confirmed that 9- and 11-year-olds have some sense of how to process the magnitude of fractions (even for fractions which have not yet been covered in the school curriculum) even if this ability is still immature at the age of 9. Finally, we have shown that symbolic notation already has a positive impact on performance by the age of 11. Further research should take these results into account in order to better target the sources of the difficulties that children encounter in the processing of ratios of natural numbers and to draw relevant implications for mathematical instruction and remediation. Relying on children’s ability to approximate the magnitude of ratios, rather than focusing on the procedures for exact computation on components (such as the search of a common denominator and the computation of cross products) in tasks such as the one used here could help children to learn the meaning of fractions and to extend their conceptual and procedural knowledge of those symbolic ratios (see also 31). 

## Supporting Information

Figure S1 (TIF)

Results S1 (DOCX)

Results S2 (DOC)
