# Supplementary figures and images for: The Processing of Symbolic and Nonsymbolic Ratios in School-Age Children
Source: PLoS One. 2013 Nov 29;8(11):e82002. doi: 10.1371/journal.pone.0082002 (PMC3843730; doi:10.1371/journal.pone.0082002)

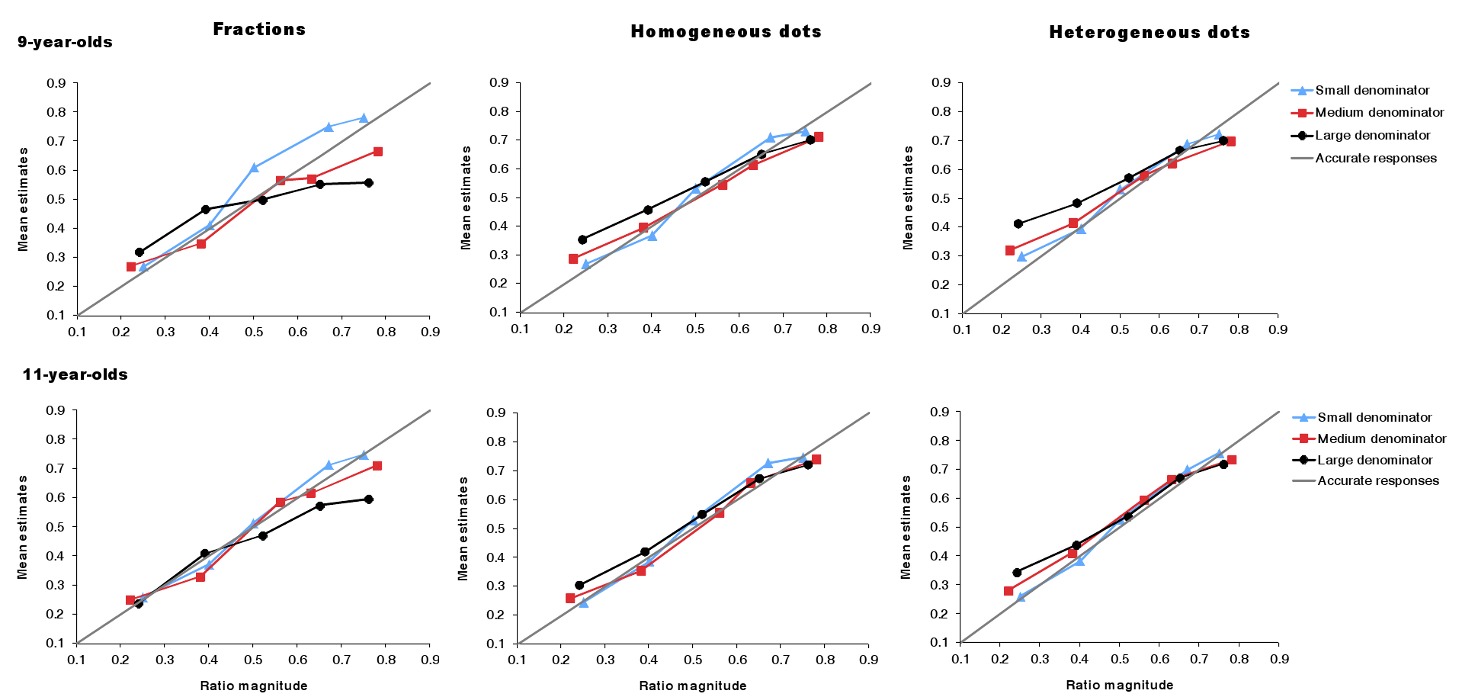

Supplement: Figure S1 — (TIF) [file pone.0082002.s001.tif]
