# Supplementary material for: The Processing of Symbolic and Nonsymbolic Ratios in School-Age Children
Source: PLoS One. 2013 Nov 29;8(11):e82002. doi: 10.1371/journal.pone.0082002 (PMC3843730; doi:10.1371/journal.pone.0082002)
Supplement: Results S1 — (DOCX) [file pone.0082002.s002.docx]

Results S1: Pattern of under- and overestimation (ANOVA on the error score)

Psychophysics studies on the judgment of proportions showed that adults tend to overestimate ratios under the half and underestimate ratios above the half (if the Steven exponent is smaller than 1, which is determined by the sensory continuum to be judged) or the reverse (if this exponent is larger than 1) [e.g., 42, 48], and that these biases increase with the size of the components [42]. We ran an ANOVA on the error score (ES) to test whether the same pattern of biases applied to the school-age children’s estimates as well. The ANOVA included Ratio Magnitude (two levels: ratios smaller vs. larger than the half), Ratio Format (three levels: fractions, homogeneous dots, heterogeneous dots) and Denominator Size (three levels: small, medium, large) as within-participants variables, and the age group as a between-participant variable. Only error scores for ratios on both sides of ~ .53 were included into this analysis (i.e., ratios with a magnitude of ~ .22, ~ .40, ~ .65 and ~ .75).

The main effect of the age group was not significant, *F*(1, 34) < 1, *p* > .10. The main effects of Ratio Magnitude and Format were significant, respectively *F*(1, 34) = 18.86, *p* < .01, η^2^ = .36 and *F*(2, 68) = 13.93, *p* < .01, η^2^ = .29, whereas the main effect of Denominator Size was not significant, *F*(1.22, 41.36) = 2.56, *p* = .11. Only the effect of Ratio Magnitude significantly interacted with the age group, *F*(1, 34) = 4.14, *p* = .05, η^2^ = .11. All the double interactions were significant [Ratio Magnitude * Ratio Format: *F*(2, 68) = 4.50, *p* = .02, η^2^ = .12; Ratio Magnitude * Denominator Size: *F*(1.59, 54.09) = 86.36, *p* < .01, η^2^ = .72; Ratio Format * Denominator Size: *F*(2.83, 96.16) = 15.24, *p* < .01, η^2^ = .31]. Among them, only the interaction between Ratio Magnitude and Denominator Size significantly depended on the age group, *F*(1.59, 54.09) = 3.68, *p* = .04, η^2^ = .10. Finally, the triple interaction Ratio Magnitude * Ratio Format * Denominator Size was significant as well, *F*(2.80, 95.16) = 7.23, *p* < .01, η^2^ = .18, and did not depend on the age group, *F*(2.80, 95.16) = 1.16, *p* > .10.

The triple interaction was explained by the fact that the pattern of biases differed between the fraction condition and the two dot conditions. This explanation was supported by a non- significant triple interaction when the ANOVA was only run on error scores for dot conditions. T-tests against 0 were run in order to identify significant biases.

For fractions, significant biases were mainly found for fractions above the half. Nine-year-olds overestimated them when the denominator was small, *t*(16) = 2.41, *p* = .03, but underestimated them when the denominator was medium, *t*(16) = -4.29, *p* < .01, and large, *t*(16) = -5.52, *p* < .01(see Figure S1). Eleven-year-olds also underestimated fractions above the half when the denominator was medium, *t*(18) = -3.04, *p* < .01, and large, *t*(18) = -6.43, *p* < .01. Regarding fractions smaller than the half, 9-year-olds overestimated them only when the denominator was large, *t*(16) = 3.33, *p* < .01, while 11-year-olds did not show any significant biases (all *p*s > .05).

(Insert Figure S1 about here)

For dot sets, significant biases were only found for ratios with medium and large denominators and this was mainly for ratios under the half. Nine-year-olds overestimated ratios under the half when the denominator was medium and large [Homogeneous dots – Medium denominator: *t*(16) = 2.51, *p* = .02; Homogeneous dots – Large denominator: *t*(16) = 5.29, *p* < .01; Heterogeneous dots – Medium denominator: *t*(16) = 3.15, *p* < .01; Heterogeneous dots – Large denominator: *t*(16) = 5.40, *p* < .01]. Eleven-year-olds also overestimated ratios above the half when the denominator was medium in the heterogeneous dot condition, *t*(18) = -3.91, *p* < .01, and when the denominator was large in both dot conditions [Homogeneous dots: *t*(18) = 2.72, *p* = .01, Heterogeneous dots: *t*(18) = 5.06, *p* < .01].Regarding ratios above the half, 9-year-olds significantly underestimated them only when the denominator was medium [Homogeneous dots: *t*(16) = -3.05, *p* < .01, Heterogeneous dots: *t*(16) = -2.92, *p* = .04].

In summary, children mainly underestimated fractions above the half and overestimated ratios of dots sets under the half. In both cases, biases were obvious for ratios with medium and large denominators and increased with the size of the denominator. This pattern of bias is consistent with the pattern shown in previous studies in adults [42, 48]. This issue should nevertheless be further investigated because the pattern of bias depends on the number of reference points the participants use to perform the task. The typical one-cycle pattern (overestimation and then underestimation*)* is shown when participants only use 0 and 1 as reference points. This pattern repeats itself if participants use intermediate reference points, the number of repetitions depending on the number of reference points (see the cyclical power model suggested by Hollands and Dyre [43]). If the participants use the half as an intermediate reference point, a two-cycle pattern should appear (overestimation under 1/4, underestimation between 1/4 and 1/2, overestimation between 1/2 and 3/4, and underestimation above 3/4). We could not test whether children used intermediate reference points in the present study as we only used five levels of magnitude that did not cover all the portions of the magnitude range for which the cyclical power model makes specific predictions.

Figure Legend

**Figure S1. Mean estimates according to the ratio magnitude and the denominator size by format and by age group** (top panel: 9-year-olds, bottom panel: 11-year-olds). The grey line represents what would be accurate responses. The figure mainly shows that, when denominators were medium and large, children underestimated fractions above the half, and overestimated ratios of dots sets under the half.
