# Supplementary material for: The Processing of Symbolic and Nonsymbolic Ratios in School-Age Children
Source: PLoS One. 2013 Nov 29;8(11):e82002. doi: 10.1371/journal.pone.0082002 (PMC3843730; doi:10.1371/journal.pone.0082002)
Supplement: Results S2 — (DOC) [file pone.0082002.s003.doc]

Results S2: ANOVA on the standard deviation (SD)

An ANOVA was run on SD with Format (three levels: fractions, heterogeneous dots and homogeneous dots) and Denominator Size (three levels: small, medium and large) as repeated factors and Age Group (two levels: 9- vs. 11-year-olds) as a between-participant factor. Post hoc tests were run to explain any significant main effect or interaction. The critical comparison for assessing the impact of ratios of surface areas was the comparison of performance on homogeneous-dot sets (no control for ratios of surface areas) with that on heterogeneous-dot sets (such a control), whereas the critical comparison for assessing the effect of symbolic notation was the comparison of performance on fractions with that on heterogeneous-dot sets (condition in which children had to process ratios of natural numbers due to the control for ratios of surface areas).

The effect of Age Group was significant, *F*(1, 34) = 11.63, *p* < .01. The main effect of Format was not significant, *F*(2, 68) = 2.32, *p* > .10, but it was qualified by a significant interaction with Age Group, *F*(2, 68) = 3.33, *p* = .04. This interaction was explained by the fact that estimates were significantly more variable in 9-year-olds than in 11-year-olds only for homogeneous-dot sets and for fractions (both *p*s < .05). Neither the effect of Denominator Size, *F*(1.44, 48.84) = 1.98, *p* > .10, nor the interaction between Denominator Size and Age Group, *F*(1.44, 48.84) = 1.73, *p* > .10, were significant. The interaction between Format and Denominator Size was significant, *F*(3.29, 111.92) = 4.38, *p* < .01, and did not significantly depend on Age Group, *F*(3.29, 111.92) < 1, *p* > .10. Despite the absence of significant triple interaction, we ran a separate ANOVA for each age group given the difference in the pattern of results between these groups.

In 9-year-olds, neither the main effects nor the interaction were significant (all *p*s > .10). By contrast, in 11-year-olds, the effect of Format, *F*(2, 36) = 11.13, *p* < .01, and of Denominator Size, *F*(2, 36) = 4.71, *p* = .03, were significant as well as the two-way interaction, *F*(4, 72) = 3.79, *p* = .02. In this group, the effect of Format was significant whatever the size of the denominator [small: *F*(2, 36) = 5.57, *p* = .02; medium: *F*(2, 36) = 7.94, *p* < .01; large: *F*(2, 36) = 12.04, *p* < .01]. Regarding the impact of ratios of surface areas, the same results as those for AES were shown: estimates were more variable in the heterogeneous-dot condition than in the homogeneous-dot condition when the denominator was medium and large, *t*(18) = –3.41, *p* < .01 and *t*(18) = –5.31, *p* < .01 respectively. So, beyond the subitizing range, performance was worse when the ratio of surface areas was controlled for than when it was not controlled for. Regarding the impact of symbolic notation, when the denominator was small, estimates of fractions were less variable than estimates for both homogeneous-dot sets, *t*(18) = 2.66, *p* = .02, and heterogeneous-dot sets, *t*(18) = 4.65, *p* < .01. When the denominator was medium or large, estimates of fractions varied less than estimates for heterogeneous-dot sets [medium: *t*(18) = 3.97, *p* < .01; large: *t*(18) = 3.42, *p* < .01] and as much as estimates for homogeneous-dot sets [medium: *t*(18) = 0.41, *p* >.10; large: *t*(18) = –1.07, *p* > .10]. These results indicate less variability for fractions than for heterogeneous-dot sets regardless of the size of the denominator.
